# Comparison of Free Energy Surfaces Calculations from *Ab Initio* Molecular Dynamic Simulations at the Example of Two Transition Metal Catalyzed Reactions

**DOI:** 10.3390/ijms12021389

**Published:** 2011-02-23

**Authors:** Marc Brüssel, Philipp J. di Dio, Kilian Muñiz, Barbara Kirchner

**Affiliations:** 1 Wilhelm-Ostwald-Institute for Physical and Theoretical Chemistry, University Leipzig, Linnéstr. 2, D-04103 Leipzig, Germany; E-Mails: gurkete@gmx.de (M.B.); naphthalen@aol.com (P.J.D.); 2 Institute of Chemical Research of Catalonia (ICIQ), Av. Països Catalans, 16, E-43007 Tarragona, Spain; E-Mail: kmuniz@iciq.es; 3 Catalan Institution for Research and Advanced Studies (ICREA), Pg. Lluís Companys 23, E-08010 Barcelona, Spain

**Keywords:** thermodynamic integration, metadynamics, free energy calculation, ab initio molecular dynamics, transition metal catalysis

## Abstract

We carried out *ab initio* molecular dynamic simulations in order to determine the free energy surfaces of two selected reactions including solvents, namely a rearrangement of a ruthenium oxoester in water and a carbon dioxide addition to a palladium complex in carbon dioxide. For the latter reaction we also investigated the gas phase reaction in order to take solvent effects into account. We used two techniques to reconstruct the free energy surfaces: thermodynamic integration and metadynamics. Furthermore, we gave a reasonable error estimation of the computed free energy surface. We calculated a reaction barrier of Δ*F* = 59.5 *±* 8.5 kJ mol^−1^ for the rearrangement of a ruthenium oxoester in water from thermodynamic integration. For the carbon dioxide addition to the palladium complex in carbon dioxide we found a Δ*F* = 44.9 ± 3.3 kJ mol^−1^ from metadynamics simulations with one collective variable. The investigation of the same reactions in the gas phase resulted in Δ*F* = 24.9 ± 6.7 kJ mol^−1^ from thermodynamic integration, in Δ*F* = 26.7 ± 2.3 kJ mol^−1^ from metadynamics simulations with one collective variable, and in Δ*F* = 27.1 ± 5.9 kJ mol^−1^ from metadynamics simulations with two collective variables.

## 1. Introduction

The *n*-dimensional energy surface of a system with *n* degrees of freedom provides all information that are important for understanding the reactivity of a system. If one explores the minimum energy path from a region of interest to another, e.g., in a reaction, one can determine the energy difference. The Helmholtz free energy surface (FES) corresponds to simulations carried out in the canonical ensemble (N,V,T), which is often applied in molecular dynamics simulations. The reconstruction of the FES with different methods is one of the outstanding characteristics of molecular dynamics simulations. A valuable overview of different techniques is provided in [1]. In this work, we applied two of those techniques, namely thermodynamic integration (TDI) [2–6] and metadynamics (MTD) [7]. Both TDI and MTD have been widely employed to investigate different reactions [8–11].

**Figure 1.**
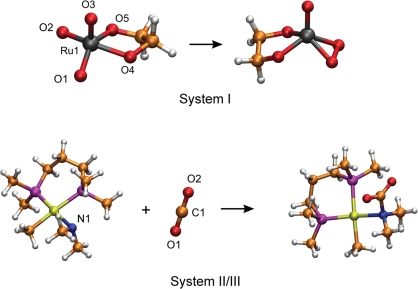
Investigated reactions. *Top*: Rearrangement of a ruthenium oxoester to a peroxoester; *Bottom*: Carbon dioxide addition to a palladium complex.

Especially in a catalytic process, the knowledge of the underlying FES is very useful when considering possible side reactions or forcing a desired reaction to take place. In this work we selected two simple reactions of transition metal complexes (Figure 1) and carried out *ab initio* molecular dynamic simulations in order to explore their FES.

**Figure 2.**
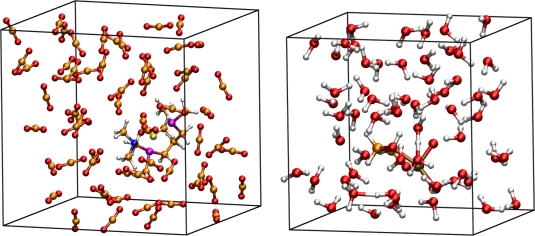
*Left*: Palladium complex with 60 carbon dioxide molecules in a solvent box (**III**); *Right*: Ruthenium complex with 60 water molecules in a solvent box (**I**).

The first reaction we treated was an intramolecular rearrangement in a ruthenium complex of two oxygen ligands to a peroxo ligand, see Figure 1. These kind of ruthenium complexes participate in the *cis*-dihydroxylation of alkenes or in the oxidative cyclization of 1,5- and 1,6-dienes [12–14]. The mechanism was previously investigated using static quantum chemical calculations [15]. Because an isomerization of the complex might make additional reaction pathways possible, we wanted to investigate this reaction including possible solvent effects with the help of *ab initio* molecular dynamics simulations. In order to achieve this, the reaction was studied in a solvent box containing 60 molecules of water (system **I**). The advantage of explicit solvent inclusion is the fact that it is not necessary to deal with possible additional solvent ligands in order to find the best transition state. In other words it is not necessary to perform several static quantum chemical calculations with different numbers of ligands. Thus the arbitrariness which arises with the choice of the computed structures in static quantum chemical calculations is removed. The second reaction considered was the intermolecular addition of carbon dioxide to a palladium complex [16]. Carbon dioxide activation is an important topic in catalysis [17,18]. There is a growing interest in reactions and catalysts which are able to activate carbon dioxide. We selected one step from the reaction which leads to a carbamate palladium complex. This reaction was investigated in vacuum (system **II**) and in a solvent box containing carbon dioxide (system **III**). We chose carbon dioxide, because it is widely applied in such syntheses. Additionally, we selected the density of the carbon dioxide to correspond to supercritical conditions. **II** represents a typical example of a substrate addition to the catalyst. One advantage of molecular dynamic simulations is the fact that the best direction for the addition to the complex is provided by the simulations. Of course, this depends on the initial starting conditions and temperature, however much more conformations are visited without any effort. This is in contrast to static quantum chemical calculations in which one has to consider all possible directions for the addition individually. Furthermore, we included the solvent molecules to investigate the effect on the stability of these kinds of complexes. In Figure 2, we show snapshots taken from molecular dynamics simulations of **II** and **III**.

Besides many advantages of molecular dynamics simulations [19], there are also several disadvantages [20,21]. For example, one has to keep in mind that in contrast to static calculations of the free energy, in simulations nuclear quantum effects [22] are usually neglected and therefore those effects are lacking in the FES.

## 2. Methodological Section

### 2.1. Computational Details

All simulations were carried out employing Born–Oppenheimer molecular dynamics simulations with Gaussian and plane waves using the program cp2k [23,24]. The temperature was set to 350 K for the ruthenium system (**I**) and to 300 Ryd K for the palladium systems (**II**,**III**) controlled by Nosé–Hoover chain thermostats [25–27]. A time step of 0.5 fs was chosen and the pseudopotentials of Goedecker, Teter and Hutter (GTH) described the core electrons of all atoms [28,29]. The DZVP basis set [30] represented the Kohn–Sham orbitals, and the plane wave representation was truncated with a cutoff of 300 for the ruthenium system and 280 Ryd for the palladium systems. The gradient corrected functional BLYP with dispersion corrections was used throughout the simulations [31–33]. For all simulations of the ruthenium system (**I**) the local spin density approximation (LSD) was applied. **I** was simulated in a periodic cubic box with 1223 pm box length containing 60 molecules of water (*ρ* = 1.17 g cm^−3^). The palladium system (**II**) without carbon dioxide solvent was simulated in a cubic box with 1500 pm box length without periodic boundary conditions. The palladium system (**III**) (*ρ* = 0.85 g cm^−3^) containing 60 molecules carbon dioxide was simulated in a periodic cubic box with 1800 pm box length. In order to obtain a starting structure for **I** and **III**, we performed molecular dynamics simulations in the framework of the program package GROMACS 3.3.1 [34]. The initial structures for the metadynamics simulations were pre-equilibrated with cp2k with Nosé–Hoover chain thermostats coupled to each vibronic degree of freedom for 0.8 ps (**II**) at 300 K and 5.5 ps (**III**) at 500 K. To allow relaxation both systems were equilibrated with a global Nosé–Hoover chain thermostats for 3 ps (**II**) and 9 ps (**III**) at 300 K. For each point of the thermodynamic integration for (**II**) and (**III**) a simulation with a constrained distance (∼9 ps for (**II**) and ∼8 ps for (**III**)) was conducted. The different simulations to obtain the constraint force for the thermodynamic integration were carried out without pre-equilibration. For the static calculations the program package TURBOMOLE 6.0 [46] was used to optimize all structures reported in this paper We applied the BP86 [32,35] functional where the RI approximation can be employed [36–38]. The def2-TZVPP [54] basis set, which includes a relativistic electron core potential for ruthenium, was used [39,40]. The program package SNF 4.0 was used for frequency calculations [41]. The figures were generated with Matplotlib which is a 2D plotting library for Python [42].

### 2.2. Thermodynamic Integration

In the thermodynamic integration approach the FES is reconstructed by integrating the negative value of the mean force with respect to the reaction coordinate *ξ* along the reaction pathway. The superscript *^′^* denotes that the conditional average is evaluated at *ξ^′^*. The reaction coordinate *ξ* is a function of the ionic coordinates **R***_i_*. The subscript *i* denotes the number of the particle.

(1) $$\Delta F=\int_{\xi_{0}}^{\xi_{1}}{\langle \frac{\partial H}{\partial \xi }\rangle }_{\xi^{\prime }}d\xi^{\prime }$$

For each integration point a simulation with a fixed value of *ξ*(**R***_i_*) is performed. The mean force can be determined from the Lagrange multiplier λ provided by molecular dynamic simulations. A general relation between the Lagrange multiplier and the conditional average is shown in Equation 2 [43]:

(2) $${\langle \frac{\partial H}{\partial \xi }\rangle }_{\xi^{\prime }}=\frac{\langle Z^{-1/2}[-\lambda +k_{B}TG]\rangle }{\langle Z^{-1/2}\rangle }$$

where *k_B_* is the Boltzmann constant and *T* is the temperature. *Z* (Equation 3) is the correction factor from the blue-moon ensemble method [5] and *G* (Equation 4 with $\xi_{x_{i}x_{j}}=\frac{\partial^{2}\xi }{\partial x_{i}\partial x_{j}}$) is a factor introduced by Sprik and Ciccotti [43] to avoid the appearance of generalized coordinates. The sum in Equation 3 and Equation 4 runs over all N ionic coordinates in *ξ*(**R***_i_*) in Equation 4. These corrections are necessary because in the molecular dynamics simulations with a fixed *ξ* = *ξ^′^* an additional constraint is introduced namely *ξ̇* = 0. The blue-moon approach corrects the additional constraint for a velocity-independent observable. Equation 2 further provides further the correct average for a velocity-dependent observable.

(3) $$Z=\sum_{i=1}^{N}\frac{1}{m_{i}}{\left(\nabla_{i}\xi \right)}^{2}$$

(4) $$G=Z^{-2}\sum_{i,j=1}^{N}\frac{1}{m_{i}m_{j}}{\left(\nabla_{j}\xi \right)}^{T}\left(\begin{array}{ccc} \xi_{x_{i}x_{j}} & \xi_{x_{i}y_{j}} & \xi_{x_{i}z_{j}}\\ \xi_{y_{i}x_{j}} & \xi_{y_{i}y_{j}} & \xi_{y_{i}z_{j}}\\ \xi_{z_{i}x_{j}} & \xi_{z_{i}y_{j}} & \xi_{z_{i}z_{j}} \end{array}\right)(\nabla_{i}\xi )$$

Equation 4 can be rewritten by introducing a 3*N* × 3*N* diagonal matrix *M* (Equation 5). And replacing the cutout of the Jacobian matrix in Equation 4 with the complete 3*N* × 3*N* Jacobian matrix *Jξ*.

(5) $$M=\text{diag}(\frac{1}{m_{1}},\;\frac{1}{m_{1}},\;\frac{1}{m_{1}},\;\frac{1}{m_{2}},\ldots \frac{1}{m_{n}})$$

This leads to a clearer relation (Equation 6), which is formally identical to Equation 4.

(6) $$G=Z^{-2}{\left(M\nabla \xi \right)}^{T}J\xi (M\nabla \xi )$$

As reaction coordinate we chose the distance between O1 and O2 for **I** and the distance between N1 and C1 for **II**, see Figure 1. In the case of a distance as reaction coordinate the relation is simple. In this case *G* becomes zero and *Z* is constant over all time steps and can be canceled in Equation 2. Thus, the average can be obtained directly from the Lagrange multiplier. We used cubic splines to interpolate the force between points obtained from simulations. How the error of the force and the FES was estimated, is described in Section 3.1.

### 2.3. Metadynamics

Within the metadynamic approach [7] a set of collective coordinates is chosen. The collective coordinates are functions of the ionic coordinates *S_m_*(**R***_i_*). For the selection of the variables two issues are important. Firstly, the variables should describe the reaction very well. Secondly, the reactants and products should be clearly distinguishable in the values of the collective coordinates. Each *S_m_*(**R***_i_*) has a corresponding collective variable (CV) *s_m_*. The idea is to explore the FES in the CV space which should describe the most important characteristics. The extended Lagrangian metadynamics method [44] was employed in this work to describe the system (Equation 7).

(7) $$\text{callL}={\text{callL}}_{0}(R_{1}..R_{N},\;{\dot{R}}_{1},..{\dot{R}}_{N})+\sum_{m}^{E}\frac{1}{2}M_{m}{\dot{s}}_{m}^{2}-\sum_{m}^{E}\frac{1}{2}k_{m}{\left[S_{m}({\text{R}}_{i})-s_{m}\right]}^{2}+V(t,\;\text{s})$$

The sums in Equation 7 run till the total number of CVs (*E*). *M_m_* denotes the mass of the *m*th CV, *k_m_* the force constant for the *m*th CV and s the vector of all CVs. In this approach the collective variables are coupled with a harmonic term to the Lagrangian of the system. Additionally, a history dependent potential is added *V*(*t*, s). The harmonic term ensures that the value of the CV *s_m_* stays close to the collective coordinate *S_m_*(**R***_i_*). In our case the functional form of the history dependent potential is a sum of Gaussians along the trajectory of the CVs (Equation 8). *H* stands for the height of the Gaussians and $\Delta s_{m}^{⊥}$ denotes the width. *P*(*t*) stands for the upper limit of the sum of Gaussians, which is time dependent.

(8) $$V(t,\;s)=\sum_{j}^{P(t)}H\prod_{m}^{E}\text{exp}\left\{-\frac{1}{2}{\left(\frac{s_{m}-s_{mj}}{\Delta s_{m}^{⊥}}\right)}^{2}\right\}$$

Because of the history dependent potential, points which were already visited in the CVs space become less favorable. This leads to a fast exploration of the FES. The most valuable asset is the possibility to reconstruct the FES in the CV space from the Gaussian hills added continuously during the simulations. The important parameters in metadynamics simulations are: *H*, $\Delta s_{m}^{⊥}$, *M_m_*, *k_m_* and the time step *τ* between spanning two Gaussians. In order to achieve a reasonable MTD run, the parameters have to be adjusted to the investigated system. In the simulations we chose *H* = 0.788 kJ mol^−1^ and for the time interval step *τ* = 0.2 ps. The parameter for the C1–N1 bond are: $\Delta s_{1}^{⊥}=400\;\text{pm}$, *M*_1_ = 100 amu and *k*_1_ = 0.075 kJ mol^−1^ pm^−2^. For the O1–C1–O2 angle: $\Delta s_{2}^{⊥}=0.3\;\text{rad}$, *M*_2_ = 100 amu and *k*_2_ = 2362.950 kJ mol^−1^ rad^−2^. The choice of the parameter is mainly based on the factors given in [45]. The error for the MTD runs were estimated with the help of Equation 9 [46]:

(9) $$\bar{ɛ}=C(d)\sqrt{\frac{k_{B}THS\Delta s^{⊥}}{\tau D}}\left(1+40\frac{\tau }{\tau_{s}}\right)$$

*S* denotes the size of the system, *C*(*d*) is a factor depending on the dimensionality of the FES (*C*(1) = 0.5, *C*(2) = 0.3), $\tau_{s}=\frac{S^{2}}{D}$ and *D* is the diffusion coefficient. How these variables are estimated is described in Section 3.3.

## 3. Results and Discussion

### 3.1. Thermodynamic Integration of the Ruthenium Peroxo Rearrangement with a Water Box (System **I**)

**Figure 3.**
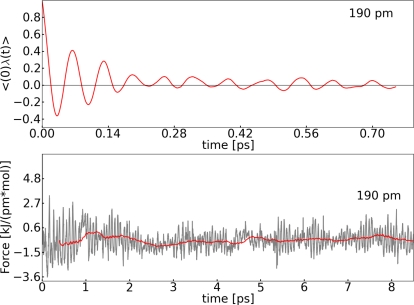
All values refer to system **I**; *Top*: Auto-correlation function of the constrained force calculated at 190 pm; *Bottom*: Value of the constrained force during the simulation (grey), running average of the constrained force *M*(*t*) (red) calculated at 190 pm.

For system **I**, the reaction coordinate (RC) was set to the distance between O1–O2. We carried out constrained simulations along the RC at selected points (140, 160, 180, 190, 210, 230 and 250 pm). The convergence of the mean force value was estimated by monitoring the running average *M*(*t*) [47]. *M*(*t*) was used to filter the variability caused by vibrations of the bond. In addition the auto-correlation function (ACF) of the constrained force was calculated to demonstrate the fast relaxation time compared to the simulation time. Figure 3 shows the values calculated for a selected example (190 pm) of the O1–O2 distance. The curves calculated for the other points show approximately the same characteristics.

Additionally, we analyzed the convergence of the observable by evaluating the difference between two successive values of the running average *M*(*t*) (Equation 10). *I* stands for the size of the time interval between two successive values. In our case *I* = 0.5 fs.

(10) G(n)=M(I(n+1))−M(I(n))

**Figure 4.**
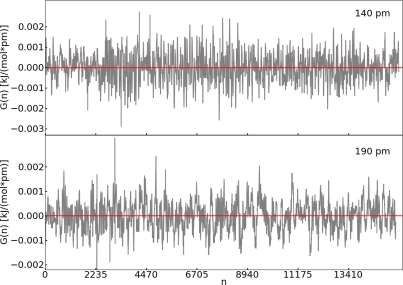
All values refer to system **I**; *Top: G*(*n*) (grey) and average of *G*(*n*) (red) calculated at 140 pm; *Bottom: G*(*n*) (grey) and 〈*G*(*n*)〉 (red) calculated at 190 pm (system **I**).

Figure 4 shows the trend of *G*(*n*) for two selected examples (140 pm and 190 pm). The value of *G*(*n*) fluctuates around zero. The deviation of the average of *G*(*n*) from zero is very small and negligible compared to the values of the mean force (Table 1). Therefore, we consider the value of *G*(*n*) as a good criterion in order to judge the convergence of the mean force. Convergence is reached if 〈*G*(*n*)〉 is below the arbitrary value of 1 × 10^−4^ kJ pm^−1^ mol^−1^ h.

**Figure 5.**
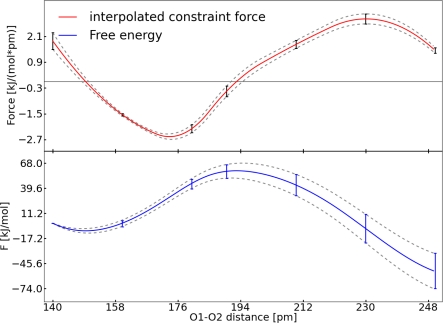
All values refer to system **I**; *Top*: Interpolated constrained force (red), interpolated upper and lower error functions (grey); *Bottom*: FES (blue), error of the FES (grey).

The average of *M*(*t*) provides the final value for the mean force. The error bars of the constrained simulations points were estimated from the standard deviation of *M*(*t*). The standard deviation of *M*(*t*) is set as the upper and lower bound of the error. The points were interpolated with the help of cubic splines (Figure 5).

The negative of the interpolated function was integrated to obtain a value for the barrier. The error of the constructed FES was estimated by integrating the upper and lower error curves of the force and subtract them from each other. The barrier which was determined in this way is Δ *F* = 59.5 ± 8.5 kJ mol^−1^. Thus the barrier is small enough that the reaction can take place at the selected conditions. To compare the order of magnitude of this barrier we carried out static quantum chemical calculations to determine the transition state of the ruthenium complex without any additional solvent molecule. We received a free energy difference of Δ *F* = 93.8 kJ mol^−1^. This difference is reasonable, considering the different methods and the lack of any additional solvent effect in the static calculations. The structure of the ruthenium peroxoester (educt), the transition state and the ruthenium oxoester (product) is compared in Table 2. The transition state is located at a O1–O2 distance of approximately 192 pm. Hence the simulation at 190 pm was used to characterize the transition state. The simulations at 140 and 250 pm were used to characterize the ruthenium peroxoester and the ruthenium oxoester.

**Figure 6.**
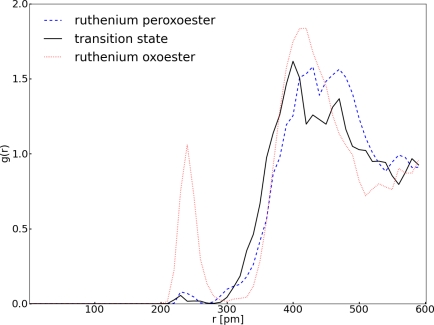
All values refer to system **I**; *g*(*r*) denotes the RDF and *r* is the distance between Ru1 and the oxygen atoms of the water.

The structure of the transition state is very similar to the ruthenium peroxoester. There are no significant changes in the structure of the ruthenium complex. Another question concerning the structure of the transition state can be answered. Is there any water coordinated to the ruthenium complex in the transition state in order to lower the barrier? To answer this question the radial distribution function (RDF) of ruthenium and the oxygen atoms of the water was computed for the ruthenium peroxoester, the transition state and the ruthenium oxoester (Figure 6).

¿From the RDF it becomes clear that only negligible coordination of water to the ruthenium in the transition state is observed. This result corresponds to the fact that the transition state has almost the same structure as the ruthenium peroxoester. However, in the ruthenium oxoester water is coordinated to the ruthenium around 240 pm which implies a much stronger coordination between water and ruthenium. When changing from the peroxo to the oxorutheniumester, the formal oxidation state of Ru changes from +VI to +VIII. The minor water coordination of the peroxoester reflects that the coordination is weaker or much more hindered.

### 3.2. Thermodynamic Integration of Carbon Dioxide Addition to the Palladium Complex without Solvent (System **II**)

In this section, we employ the thermodynamic integration approach to investigate a reaction with a low barrier. The same reaction was investigated with the help of metadynamics (Section 3.3). System **II** was chosen for the TDI. We selected a distance for the reaction coordinate, namely the N1–C1 bond, see Figure 1. Constrained simulations were carried out at 165, 180, 200, 230, 250 and 290 pm. Like for the ruthenium system, we calculated the same quantities to assess convergence of the mean force. We observed the same behavior as for system **I** for each quantity. The ACF shows fast relaxation, *G*(*n*) fluctuates around zero and 〈*G*(*n*)〉 propagates close to a value of zero.

**Figure 7.**
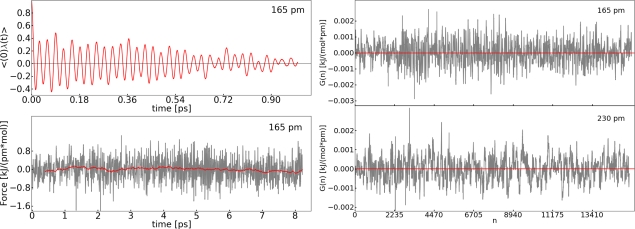
All values refer to system **II**; *Top left*: ACF of the mean force (red) calculated at 165 pm; *Top right: G*(*n*) (grey) and 〈*G*(*n*)〉 (red) calculated at 165 pm; *Bottom left*: Value of the constrained force during the simulation (grey), running average of the constrained force *M*(*t*) (red) calculated at 165 pm; *Bottom right: G*(*n*) (grey) and 〈*G*(*n*)〉 (red) calculated at 230 pm.

**Figure 8.**
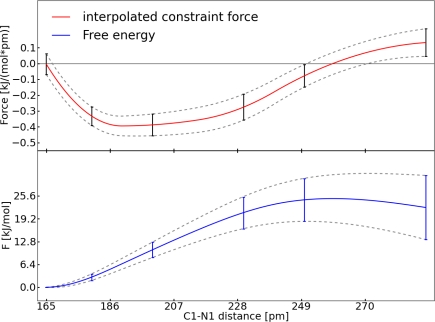
All values refer to system **II**; *Top*: Interpolated constraint force (red), interpolated upper and lower error functions (grey); *Bottom*: FES (blue), error of the FES (grey).

It should be noted that the remaining oscillation of the auto-correlation function (Figures 3 and 7) is caused by the intramolecular vibration of the bond. Additionally, the sharp peaks in the diagrams of *G*(*n*) and the mean force (Figures 3, 4 and 7) are due to Brownian motion. The FES was constructed in the same manner as mentioned above. In Figure 8 the interpolated force and the resulting FES are shown. The barrier for the addition of carbon dioxide calculated with TDI amounts to Δ*F* = 24.9 ± 6.7 kJ mol^−1^. The transition state for this reaction is found at the C1–N1 distance of 259 pm. Comparing the FES in Figures 5 and 8 the different shapes of both curves stand out. This can be explained by the fact, that system **I** changes between two stable species (ruthenium oxoester to a peroxoester). However, in system **II** only a dissociation takes place and this leads to a flat decay in the FES.

### 3.3. Metadynamic Simulations of the Carbon Dioxide Addition to the Palladium Complex without Solvent (system **II**) and one Collective Variable

We investigated the reaction for system **II** applying one collective variable, namely the C1–N1 distance, see Figure 1. To produce reasonable metadynamics simulations Equations 11 and 12 were used [45]. First *k_m_*, $\Delta s_{m}^{⊥}$ and *M_m_* were determined by simulations without a history dependent potential. Additionally the value on the left hand side of Equation 11 has to be smaller than the standard deviation of the collective coordinate. The ratio between *k_m_* and *M_m_* was adjusted to ensure adiabatic decoupling. Subsequently, the height was selected to be small compared to the depth of the well. Furthermore, the maximum force $f_{m}^{max}$ due to a Gaussian was estimated with the help of Equation 12 and compared to the force *F_S_m__*(**R***_i_*) on *S_m_*(**R***_i_*) in simulations without a history dependent potential.

(11) $$\langle {\left(s_{m}-S_{m}({\text{R}}_{i})\right)}^{2}\rangle =\frac{k_{B}T}{k_{m}}$$

(12) $$f_{m}^{\text{max}}=\frac{H}{\Delta s_{m}^{⊥}}\text{exp}\left\{-\frac{1}{2}\right\}$$

(13) $$\tau =\frac{3}{2}H\sqrt{\frac{M_{m}}{k_{B}T_{m}}}$$

*τ* was finally estimated with the help of Equation 13. Table 3 shows the values for the set of the parameters (**set I**) used in all metadynamics simulations. As an example, **set I** is compared to another set of parameter (**set II**) values for which the force constant was too low and the deviation of the CV from the collective coordinate became too large. We would like to point out that this criterion is not the only one which has to be fulfilled. For example, a too large force constant holds the CV close to the collective coordinate but destroys the adiabatic decoupling of the systems.

**Figure 9.**
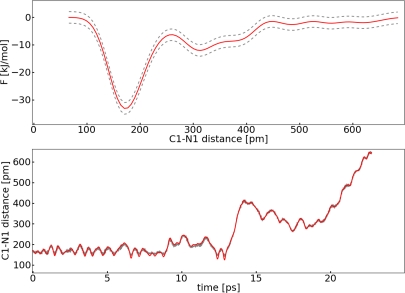
All values refer to system **II**; *Top*: Reconstructed FES of the metadynamic run with **set I** (red), interpolated upper and lower error functions (grey); *Bottom*: Dynamic of the collective variable *s*_1_ (red) and collective coordinate *S*_1_(**R***_C_*_1−_*_N_*_1_) (grey).

The FES reconstructed by the added up Gaussians, the time development of *S*_1_(**R***_C_*_1−_*_N_*_1_), and the corresponding CV *s*_1_ are shown in Figure 9. Around 13 ps the potential well is completely filled up with Gaussians and the C1–N1 bond breaks.

**Figure 10.**
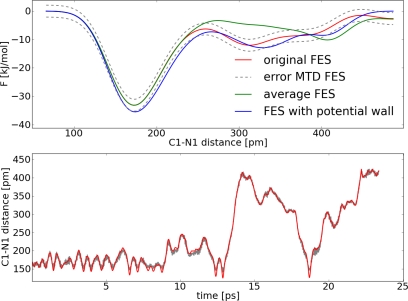
All values refer to system **II**; *Top*: Reconstructed FES of the metadynamic run with **set I** (red), average FES (green), FES with potential wall (blue), interpolated upper and lower error functions (grey); *Bottom*: Dynamic of the collective variable *s*_1_ (red) and collective coordinate *S*_1_(R*_C_*_1−_*_N_*_1_) (grey) for the simulation with a potential wall.

The error of the FES was estimated with Equation 9. *S* was set to 476 pm, which is approximately the size of the system explored within the simulation. The diffusion coefficient *D* was determined by integration of the ACF of the CV velocities (*D* = 6.610×10^3^ pm^2^ ps^−1^). Thus, we obtained a free energy difference of Δ*F* = 26.7 ± 2.3 kJ mol^−1^. This value is almost 2 kJ mol^−1^ higher compared to the TDI result. Most likely the TDI calculation underestimates the barrier, because TDI can only reproduce the right FES if the correct reaction coordinate is selected. The distance itself is a good first approximation, but it lacks some important contributions to the mean force. In contrast to calculations from the TDI with the MTD approach one tries to explore the FES with a set of limited variables. Therefore, the same choice of the geometric parameter for the reaction coordinate and the collective variable might result in different outcomes. For the carbon dioxide addition the distance itself is good enough to ensure a reasonable exploration of the FES in the MTD approach. Furthermore, we will see in the next section, that it is possible for this system to estimate satisfyingly the well depth in the FES with this one collective variable, even if there are other important variables for the reaction coordinate. An important question in the MTD approach concerns the convergence of the FES. In other words, when were enough hills added to describe the FES satisfyingly. Normally a simulation is regarded as converged if the forward and backward reaction were observed several times. There are methods like well-tempered metadynamics which avoid this problem [48]. However we will not use this method here. In Figure 9 *bottom* one can see that there are only very weak fluctuations of the CV after the first reaction occurred (*ca*. 14 ps). Since the carbon dioxide dissociates during the reaction it is difficult to observe forward and backward reactions multiple times. We explored two ways to enhance the sampling quality of the FES (Figure 10). For the first approach three points around 14 ps from the trajectory were selected. Afterwards the velocities of the atoms of the carbon dioxide were newly initialized according to the Boltzmann distribution and a MTD run was performed. The resulting free energy surfaces of the four MTD simulations were averaged. In the second approach a potential wall was used, which inverts the velocity of the CV if the distance of C1–N1 is greater than 400 pm.

With the help of the potential wall it is possible to force the backward reaction. One can observe this in Figure 10 *bottom*. For the free energy difference of the average FES (Figure 10 *top* green curve) we obtained Δ*F* = 29.8 kJ mol^−1^. Simulating with the potential wall (Figure 10 *top* blue curve) led to a free energy difference of Δ*F* = 28.2 kJ mol^−1^. The three values of the free energy differences are close together and within the error and therefore we consider the MTD as close to being converged.

### 3.4. Metadynamics Simulations of the Carbon Dioxide Addition to the Palladium Complex without Solvent (System **II**) and Two Collective Variables

The CVs should describe the reaction as good as possible, thus we try to find additional important CVs. As the angle bends itself strongly during the reaction, it seemed to be meaningful to add the O1–C1–O2 angle to our set of CVs.

**Figure 11.**
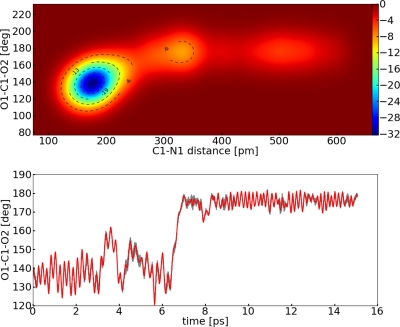
All values refer to system **II**; *Top*: Reconstructed FES of the metadynamic run in kJ mol^−1^; *Bottom*: Dynamic of the collective variable *s*_2_ (red) and collective coordinate *S*_2_(O1–C1–O2) (grey).

**Figure 12.**
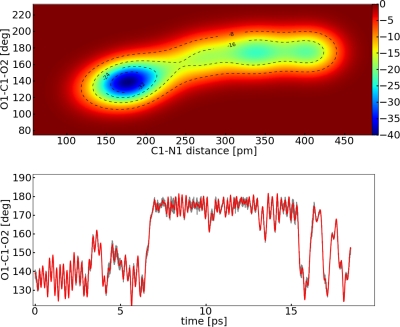
All values refer to system **II**; *Top*: Reconstructed FES of the metadynamic run with a potential wall in kJ mol^−1^; *Bottom*: Dynamic of the collective variable *s*_2_ (red) and collective coordinate *S*_2_(O1–C1–O2) (grey).

Figure 11 provides a clearer picture of the energy surface and the mechanism of the reaction. It follows that for the reaction coordinate not only the change in the distance but also the arrangement of the angle is crucial. In the minimum energy path an enlargement of the distance and simultaneously of the angle happens, see Figure 11 *top*. Again, the error of the FES was calculated with Equation 9. Due to the fact that we introduced two variables, the diffusion coefficient is actually a 2 × 2 tensor. Additionally, the scaling factor $\Delta s_{m}^{⊥}$ of the Gaussians is different. To use Equation 9 we estimated *D*, *S*, and $\Delta s_{m}^{⊥}$ with the help of the geometric mean (*D* = 12.1 rad pm ps^−1^, *S* = 22 rad^−0.5^ pm^−0.5^ and $\Delta s_{m}^{⊥}=2.5\;{\text{rad}}^{-0.5}{\text{pm}}^{-0.5}$). We obtained Δ*F* = 27.1 ± 5.9 kJ mol^−1^. Since only one reaction event was observed in the simulation, again an additional potential was used to improve the sampling of the FES (Figure 12). The potential wall was placed again at 400 pm.

Simulating with the potential wall, Δ*F* = 23.8 kJ mol^−1^ was obtained. The multiple occurrence of the reaction leads to a better sampling of the FES and a slightly lower value was obtained. Hence the initial metadynamics run was not completely converged. However, the value is in the error range, therefore we use the first value for the discussion. A final remark should be given to the additional minima in the FES obtained from a MTD run, e.g., in Figure 10 around 320 and 400 pm. These minima are obviously artifacts of the simulations. They are simply a result of the bumpy reconstruction of the FES.

### 3.5. Metadynamic Simulations of the Carbon Dioxide Addition to the Palladium Complex with a Carbon Dioxide Box (System **III**) and One Collective Variable

**Figure 13.**
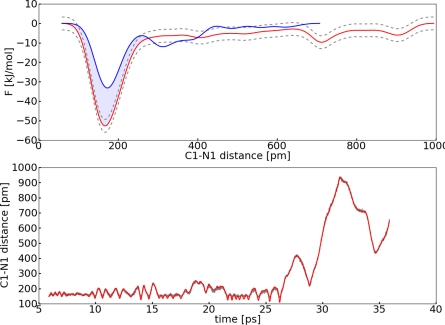
All values refer to system **III**; *Top*: Reconstructed FES of the metadynamic run for system **II** (blue), interpolated upper and lower error functions (grey), reconstructed FES of the metadynamic run for system **I** (red); *Bottom*: Dynamic of the collective variable *s*_2_ (red) and collective coordinate *S*_1_(**R***_C_*_1−_*_N_*_1_) (grey).

In order to evaluate solvent effects we simulated the reaction with a solvent box of carbon dioxide as well. For the metadynamics simulations the same parameters were used as in system **II** (with one CV). Comparing both FESs the effect of the solvent becomes obvious (Figure 13). We observe an increase in the barrier. Both enthalpic and entropic contributions of the solvent may be important for this effect. It is of course impossible to derive a quantitative statement from the total FES about the order of magnitude of these different contributions. However, one can infer, at least qualitatively, the mechanism of the stabilizing contributions.

**Figure 14.**
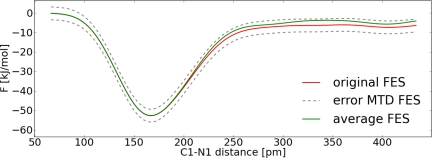
All values refer to system **III**; Average FES (green), interpolated upper and lower error functions (grey), reconstructed FES of the metadynamic run for system **I** (red).

It is obvious that additional favorable interactions with additional solvent molecules can lead to stabilizing effects. If these contributions are only occurring at the educt state or if the transition state is destabilized then the enthalpic effect from the solvent would lead to a higher reaction barrier. Additionally, if one compares **II** to system **III**, the change in entropy caused by the dissociation of the carbon dioxide becomes less important in **III**. In other words, the entropy might increase less, which leads to a stabilization of the palladium carbon dioxide complex. The barrier of the dissociation in the solvent amounts to Δ*F* = 44.9 ± 3.3 kJ mol^−1^ (*D* = 5.3 × 10^3^ pm^2^ ps^−1^). The solvent effects contribute 17.8 kJ mol^−1^ to the stabilization of the complex. This represents approximately 65% of the binding energy in vacuum. To enhance the sampling of the FES we used the same approach as in Section 3.3, *i.e.*, and selected three points around 30 ps, initialized new velocities for the atoms of the carbon dioxide and averaged over the four FES (Figure 14). A free energy difference of Δ*F* = 46.6 kJ mol^−1^ was obtained. Since both values are of the same order of magnitude, we assume that the simulation is converged.

## 4. Summary and Conclusions

With the aid of the thermodynamic integration (TDI) approach we learned the following about the reactions of the ruthenium complex. For the intramolecular rearrangement of the ruthenium complex with solvent water we obtained a barrier of Δ*F* = 59.5 ± 8.5 kJ mol^−1^. This leads to the conclusion that this reaction can take place in solution. Therefore, both species (educt and product) can be present in this system and, if specific reactions are investigated, one should consider all possible reactions for both species. This can lead to undesirable side reactions, if the total activation barrier of a desired reaction is much higher than the barrier of the interconversion of the two complex isomers. Furthermore, it was possible to show that there is no water directly bonded to the ruthenium in the transition state and that the transition state has a very similar structure than the ruthenium peroxoester.

**Figure 15.**
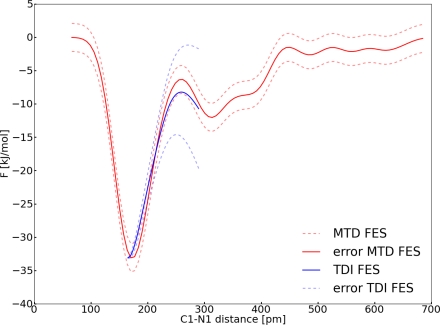
All values refer to system **II**; Reconstructed FESs of system **II**. FES from MTD (red), FES from TDI (blue).

For the carbon dioxide addition, the two free energy surfaces (FES) from the TDI and the metadynamics (MTD) approach were compared (Figure 15).

With the TDI approach the barrier amounts Δ*F* = 24.9 ± 6.7 kJ mol^−1^ and is very close (∼2 kJ mol^−1^) to the MTD run (Δ*F* = 26.7 ± 2.3 kJ mol^−1^) for the gas phase reaction. We obtained almost the same energy barrier if we simulated with either one (distance) or two (distance and angle) collective variable(s) (Δ*F* = 26.7 ± 2.3 kJ mol^−1^ and Δ*F* = 27.1 ± 5.9 kJ mol^−1^) employing MTD, although it is obvious from Figure 11 that for the minimum energy path both the angle and the distance are important. These observations can be explained in the following way. The angle is a fast degree of freedom compared to the distance in the simulation, therefore the angle can be considered at equilibrium during one MTD run. There are similar examples known in literature [49]. In the view of the reaction coordinate we found that it is important to include the angle in the RC, because both distance and angle change simultaneously during the reaction. In the TDI approach, we underestimated the barrier because we neglected the angle in the RC and the contribution to the constraint force. In the view of the set of collective variables, the distance variable fulfilled the conditions for the set of the CV satisfyingly (Section 2.3). Furthermore, the difference between the different sets of CVs (∼0.5 kJ mol^−1^) is too small to be significant compared to the estimated errors. Another important reason for the small differences between the two sets of CVs might have originated in the flat well of the FES. In conclusion, one can say that the barrier of this reaction was assessed nearly equal for one or two CVs. However, the inclusion of the angle is mandatory if a deeper insight in the reaction mechanism is desired. Finally, the influence of a solvent was investigated. The calculated binding energy of carbon dioxide to the palladium complex is very low but it rises if solvent effects are taken into account (Δ*F* = 44.9 ± 3.3 kJ mol^−1^). Hence, solvent effects have an enormous contribution to the stabilization, most likely of the carbon dioxide adduct. Therefore, one can conclude that in a catalytic process the solvent can stabilize the educt or product species and makes a subsequent reaction more difficult. In other words, if there are energetically favorable paths for the product of the carbon dioxide addition calculated without any solvent effects, they can be inaccessible in a real system.

## Figures and Tables

**Table 1.** All values refer to system **I**; Comparison of the average value of *M*(*t*) and the average value of *G*(*n*).

**Distance (pm)**	**〈*M*(*t*)〉 (kJ pm^−1^mol^−1^)**	**〈*G*(*n*)〉 (kJ pm^−1^mol^−1^)**
140	1.9	5.6 × 10^−5^
160	−1.5	−1.9 × 10^−6^
180	−2.2	−3.6 × 10^−5^
190	−0.5	3.2 × 10^−6^
210	1.7	8.6 × 10^−5^
230	2.9	9.5 × 10^−6^
250	1.4	5.5 × 10^−6^

**Table 2.** All values refer to system **I**; Average values for distances and angles of the ruthenium complex, a given O1–O2 distance. (ruthenium peroxoester 140 pm, transition state 190 pm, ruthenium oxoester 250 pm).

**Distance (pm)**	**Ru1–O1**	**Ru1–O2**	**Ru1–O3**	**Ru1–O4**	**Ru1–O5**
Ruthenium peroxoester	194	195	168	192	192
Transition state	190	193	169	191	193
Ruthenium oxoester	172	172	174	202	201

**Angle (deg)**		**O4–Ru1–O5**	**O1–Ru1–O3**	**O2–Ru1–O3**	
Ruthenium peroxoester		84	119	118	
Transition state		83	114	113	
Ruthenium oxoester		77	105	105	

**Table 3.** All values refer to system **II**; Comparison between selected sets of metadynamics parameters. The values were obtained from test simulations (2 ps) without adding Gaussians. **Set I** is calculated with the parameter $\Delta s_{1}^{⊥}=400\;\text{pm}$, *M*_1_ = 100 amu and *k*_1_ = 0.075 kJ mol^−1^ pm^−2^ and *τ* = 0.2 ps. **Set II** with the same parameters except *k*_1_ = 9 × 10^−4^ kJ mol^−1^ pm^−2^.

	〈(*s_m_* − *S_m_*(**R***_i_*))^2^〉 (pm^2^)	〈(*S_m_*(**R***_i_*) − 〈*S_m_*(**R***_i_*)〉)〉 (pm^2^)	$\frac{k_{B}T}{k_{m}}$ (pm^2^)	$f_{m}^{max}$ (kJ mol^−1^ pm^−1^)	*F_S_m__* (kJ mol^−1^ pm^−1^)
**Set I**	25.4	44.5	33.3	7.5 × 10^−3^	9.5 × 10^−1^
**Set II**	57.6	51.9	2659.1	7.5 × 10^−3^	7.5 × 10^−1^
